# Horizontal gene transfer and recombination in Streptococcus dysgalactiae subsp. *equisimilis*

**DOI:** 10.3389/fmicb.2014.00676

**Published:** 2014-12-15

**Authors:** Celia L. McNeilly, David J. McMillan

**Affiliations:** ^1^Bacterial Pathogenesis Laboratory, QIMR Berghofer Medical Research Institute, Herston, QLD, Australia; ^2^Inflammation and Healing Research Cluster, School of Health and Sport Sciences, University of the Sunshine Coast, Maroochydore, QLD, Australia

**Keywords:** *Streptococcus dysgalactiae* subsp. *equisimilis*, *Streptococcus pyogenes*, horizontal gene transfer, recombination, M-protein, bacteriophage, integrative conjugative element

## Abstract

*Streptococcus dysgalactiae* subsp. *equisimilis* (SDSE) is a human pathogen that colonizes the skin or throat, and causes a range of diseases from relatively benign pharyngitis to potentially fatal invasive diseases. While not as virulent as the close relative *Streptococcus pyogenes* the two share a number of virulence factors and are known to coexist in a human host. Both pre- and post-genomic studies have revealed that horizontal gene transfer (HGT) and recombination occurs between these two organisms and plays a major role in shaping the population structure of SDSE. This review summarizes our current knowledge of HGT and recombination in the evolution of SDSE.

## INTRODUCTION

*Streptococcus pyogenes* (group A *Streptococcus*, GAS) and *Streptococcus dysgalactiae* subsp. *equisimilis* (SDSE, human group C and G streptococci) are the two most common β-hemolytic streptococci recovered from humans. Along with *Streptococcus canis*, a bacterium causing infections in dogs and sometime humans (Galperine et al., 2007) they form a separate phylogenetic branch of the β-hemolytic streptococci (Facklam, 2002; Lefebure et al., 2012). GAS is a major pathogen of humans, and the cause of approximately half a million deaths each year. Diseases associated with GAS infection range from self-limiting throat and skin infections to the potentially fatal rheumatic fever (RF), rheumatic heart disease (RHD), and serious invasive diseases. In contrast, SDSE is generally of lesser virulence, and was once considered to be an opportunistic pathogen. Multiple studies have conclusively shown, that with the exception of RF/RHD, infection with this organism can result in elaboration of the same diseases as caused by GAS infection (Efstratiou, 1997; Chhatwal et al., 2006; O’Loughlin et al., 2007; Broyles et al., 2009). Circumstantial evidence for a link between SDSE and RF has also been provided in one instance (Haidan et al., 2000). With the similarities in disease association, colonization of the same host niches (i.e., throat and skin), and close evolutionary relationship it is unsurprising that these two species possess homologous virulence genes. Indeed, many of the classical virulence genes of GAS, such as the *emm*-genes and streptokinase, are also present in SDSE. While such genes are mostly paralogs inherited from a common ancestor, there is evidence of the transfer of virulence genes, and indeed house-keeping genes between the two species. In GAS, the acquisition of virulence genes by horizontal gene transfer (HGT) is associated with changes in virulence of some lineages (Cole et al., 2011), and this may also be true for SDSE. This review provides a concise summary of our current knowledge of HGT and recombination involving SDSE.

## SDSE VIRULENCE FACTORS AND HGT

### M-PROTEIN

The M protein, encoded by the *emm*-gene, is a critical virulence factor of GAS that is also present in SDSE. Variations in the gene corresponding to the amino-terminal part of the M-protein are also used to classify individual GAS and SDSE isolates into *emm*-types. More than 200 GAS *emm*-types and 50 separate SDSE *emm*-types have been described (Beall et al., 1996; Cohen-Poradosu et al., 2004). As these *emm*-types are species specific, their presence in a different species, or in different genetic backgrounds of the same species can be used to infer HGT involving this gene. The first such indications of interspecies HGT were provided by Maxted and Potter (1967), who showed cross-reactivity between a group G streptococcal isolate and typing sera raised against the GAS M12 protein. Several decades later the first genetic evidence for HGT of the *emm*-gene was presented (Simpson et al., 1987, 1992; Sriprakash and Hartas, 1996). More recently, multi-locus sequence typing (MLST) studies have provided new insights into extent of both inter and intra-species *emm*-gene HGT in SDSE (Ahmad et al., 2009; McMillan et al., 2010, 2011). By examining genetic differences in house-keeping genes that theoretically do not undergo HGT, MLST provides a mechanism for assessing the evolutionary relationships between clonal and distantly related isolates of the same species. Overlaying *emm*-type information onto this MLST data provides a means to predict instances of both intra-species and inter-species HGT that is not feasible using older techniques (Enright et al., 2001). Using this approach, HGT of the *emm*-gene in SDSE has been revealed to be much more common than predicted, with three separate population studies reporting the presence of the same SDSE *emm*-gene in distantly related SDSE isolates or multiple *emm*-genes in the same genetic background (Ahmad et al., 2009; McMillan et al., 2010, 2011). GAS *emm*-genes genes were also present in some SDSE isolates in these studies. While *emm*-gene HGT has also been reported in GAS (Bessen et al., 2008), it appears to be more frequent in SDSE. Together the data suggest that some caution must be taken when using *emm*-sequencing to imply genetic relatedness of SDSE isolates that are temporally or geographically unrelated.

The results of these studies open questions as to how and why such events are occurring. Using bioinformatics analyses, Panchaud et al. (2009) predicted the *emm*-gene to be part of an ancient pathogenicity island consisting of approximately 40 genes in GAS. The group went on to identify a natural isolate specifically lacking this region, and also demonstrated deletion of the region in laboratory, providing *in vitro* support for their bioinformatic findings. It is tempting to suggest that the *emm*-gene in SDSE is also part of the same pathogenicity island. Indeed a genomic comparison shows evidence for a homologous mobile genetic element (MGE) in SDSE. However, the *emm*-gene is not part of this island, and is located in a distant part of the chromosome (Suzuki et al., 2011). Thus while SDSE may acquire GAS *emm*-genes through a mechanism involving this pathogenicity island, it cannot be invoked as a hypothesis of how SDSE *emm*-genes are distributed through the population.

In GAS, the M protein is both an inhibitor of opsonophagocytosis and an adhesin (Bessen et al., 1989; Cunningham, 2000). These attributes are achieved through the protein’s ability to act as a ligand for multiple extracellular matrix and immune-system-associated proteins including fibronectin, fibrinogen, albumin, collagen, antibody, and C4b binding protein (Horstmann et al., 1988; Akesson et al., 1994; Johnsson et al., 1996, 1998; Cue et al., 2001; Dinkla et al., 2003). Apart from opsono-inhibitory activity, not all M-proteins possess all these functions, which may be a factor in the niche specialization of some GAS *emm*-types. SDSE M-proteins are known to inhibit opsonophagocytosis (Bisno et al., 1987) and reported to bind collagen (Barroso et al., 2009). The latter property has been suggested to be involved in the pathogenesis of rheumatic heart fever following GAS infection (Dinkla et al., 2007). Based on data from multiple GAS M-proteins we would not expect all SDSE proteins to have the same ligand binding properties, and switching of *emm*-genes from one to another may be a strategy for attainment of new virulence properties. However, we have no evidence that expression of different M-proteins by SDSE alters pathogenesis of individual lineages (Ikebe et al., 2004; Pinho et al., 2006). As the amino-terminus of the M-protein of GAS is also the target of *emm*-type specific bactericidal antibodies (Beachey et al., 1981; Beachey and Seyer, 1986), switching *emm*-genes may simply be a mechanism for avoidance of *emm*-type specific antibody responses.

### OTHER VIRULENCE GENES

Horizontal gene transfer and recombination are also apparent in other SDSE virulence genes. *gfbA* and *sfbI*, genes encoding fibronectin-binding proteins in SDSE and GAS are part of the fibronectin-collagen-T antigen (FCT) locus. The locus also includes genes that encode a transcriptional regulator, pilus-associated proteins and other extracellular matrix binding proteins (Mora et al., 2005; Lizano et al., 2007). Recombination within the aromatic amino acid rich (Aro) domain of both GfbA and SfbI gives rise to so-called mosaic proteins. In several instances, these proteins include mosaic domains derived from both GAS and SDSE (Towers et al., 2004). Streptokinase (*skg*) is another SDSE gene with a mosaic structure. Streptokinase interacts with plasminogen ultimately resulting in the formation of the serine protease plasmin. Plasmin activity subsequently assists the dissemination of streptococci (Ben Nasr et al., 1994). The corresponding *ska* genes in GAS are also mosaic in nature (Kapur et al., 1995; Kalia and Bessen, 2004). Again there is evidence for interspecies transfer of domains within the streptokinase gene (Tewodros et al., 1996; Kalia and Bessen, 2004). Functionally, streptokinase variants display different plasminogen activation characteristics (McArthur et al., 2008). While variations in *ska* have also been linked to tissue tropism (Kalia and Bessen, 2004) and nephritis in GAS, no attempt to find similar associations has been attempted for SDSE. The mosaic nature of *gfbA*, *skg* and their counterparts in GAS suggest the mechanism responsible for recombination is different to that associated with *emm*-gene HGT. Nevertheless, recombination with foreign DNA can only take place after a HGT event. Parts of the FCT locus are reported to be highly similar to sequences present in genomic islands in *S. agalactiae* (Beres and Musser, 2007), providing some evidence of a potential mechanism for the transfer of FCT-associated genes in other streptococcal species. The mosaic nature of these genes resembles in some respects segmentally variable genes (SVGs; Zheng et al., 2004). SVGs contain highly variable regions within well-conserved regions, and have been suggested to account for 10–20% of some bacterial genomes. The highly variable regions are suggested to be to be involved in interaction with other molecules, and be under diversifying selection pressure. As the fibronectin-binding genes and streptokinase of streptococci also interact with host molecules, recombination in these genes may be a similar strategy for diversification of the function of these genes.

DrsG provide a contemporary example of an SDSE virulence gene that is most likely part of an MGE (Minami et al., 2011; Oppegaard et al., 2014; Smyth et al., 2014). DrsG is a homolog of streptococcal inhibitor of complement (SIC) and distantly related SIC (DRS), related proteins that are secreted by GAS. *sic* and *drs* are only present in four of the more than 200 GAS *emm*-types. In *emm*1, *emm*12, and *emm*55 GAS, *sic* and *drs* are part of the mga locus. However, in *emm*57 GAS, the gene sits between *rps*U and a gene encoding an ATP-binding cassette (ABC) transporter (Binks et al., 2003). The non-coding DNA flanking *sic* in *emm*57 is highly similar to that observed which flanks *sic* in M1 GAS, suggesting that is was acquired via HGT from *emm*1 GAS. In contrast, *drsG* has been identified in 19 of the approximately 50 SDSE *emm*-types (Minami et al., 2011). However, not all isolates within a *drsG*-positive *emm*-type possess the gene. *drs*G is also highly conserved at both the nucleotide and amino acid level across all *emm*-types, with an identity greater than 95%. The gene adjacent to *drs*G in some SDSE isolates is also an MGE-related gene (Smyth et al., 2014). The data suggests that whereas *sic* and *drs* appear to have entered the *emm*1, *emm*12, and *emm*55 GAS populations prior to segregation into specific *emm*-types, *drsG* was acquired later in the evolution of individual SDSE *emm*-types. Given the high level of identity of *drsG* in different *emm-*types it is also reasonable to hypothesize that the gene may be part of an MGE that is being transferred through the SDSE population. Functionally, DrsG is more related to DRS than SIC. Whereas SIC is an inhibitor of complement (Akesson et al., 1996; Fernie-King et al., 2001), neither DRS or DrsG have this property (Smyth et al., 2014). However, all three proteins are inhibitory to the antimicrobial activity of LL-37, a cathelicidin present on mucosal surfaces (Lai and Gallo, 2009) colonized by streptococci. Both SIC and DRS are also reported to be inhibitory to other antimicrobial peptides (AMPs; Fernie-King et al., 2004; Frick et al., 2011), properties which have yet to be tested for DrsG. Together these results suggest that inhibition of AMP activity is the major function of this family of proteins. By preventing AMP mediated killing, such activity theoretically improves the colonization success of streptococci relative GAS and SDSE lacking these genes.

### TRANSFER OF ANTIBIOTIC RESISTANCE GENES

Perhaps the HGT events in SDSE with the greatest clinical impact currently involve antibiotic resistance genes. Fluoroquinolones are a class of antibiotic proscribed for treatment of streptococcal infection that function by inhibiting DNA gyrase (*gyrA*) and topoisomerase IV (*parC*). Resistance to fluoroquinolones has been reported in several streptococcal species (Kawamura et al., 2003; Reinert et al., 2004; Powis et al., 2005; Wehbeh et al., 2005) including SDSE (Biedenbach et al., 2006; Pletz et al., 2006), and occurs due to point mutations in the quinolone resistance determining regions (QRDR) of the genes encoding these proteins or through active drug efflux mechanisms. Similar to other genes described in this review, it appears that HGT and recombination involving these genes has occurred multiple times (Pletz et al., 2006; Duesberg et al., 2008).

## HGT AND RECOMBINATION INVOLVING HOUSE-KEEPING GENES

The SDSE MLST schemes targets seven genes, glucose kinase (*gki*), glutamine transport protein (*gtr*), glutamate racemase (*murI*), DNA mismatch repair protein (*mutS*), transketolase (*recP*), xanthine phosphoribosyltransferase (*xpt*), and acetoacetyl-coathioloase (*atoB*). With the exception of *atoB* the genes used for MLST in SDSE are the same as used for the GAS MLST scheme. Analogous to the HGT involving *emm*-genes the presence of GAS MLST alleles in SDSE provides strong evidence for the transfer of these theoretically fixed non-MGE-related genes (Ahmad et al., 2009; McMillan et al., 2010, 2011). In most instances, entire allelic regions were replaced with the corresponding regions from other SDSE isolates, GAS or even *S. agalactiae*. In some instances, evidence of recombination within the alleles, similar to what has been reported for *gfbA* and streptokinase was also observed. For instance, the *gki12* and *gki4* alleles of SDSE appear to be hybrids of separate SDSE and GAS alleles. These studies also revealed that, at least for the genes used in MLST, it is recombination rather that point mutation that gives rise to diversity in the population.

The mechanisms that result in HGT of house-keeping genes in SDSE are yet to be determined. In *S. agalactiae*, transfer of large sections of chromosomal DNA has been reported (Brochet et al., 2008). They demonstrated that erythromycin marked DNA segments up to 300 kb long could be transferred from a donor to recipient group B streptococci (GBS) strains via conjugation. Moreover, they were able to demonstrate that these transfers could be initiated from multiple sites. In some instances, these transfers are directly associated with genomic islands carrying genes involved in HGT. Given that *S. agalactiae* and SDSE are relatively closely related, and the high rate of recombination, it is tempting to speculate that a similar events are occurring is SDSE. If this hypothesis is correct, it would explain both the transfer of house-keeping genes and non-MGE-related virulence genes.

## MOBILE GENETIC ELEMENTS OF SDSE

The transfer of bacteriophage between GAS and group G streptococci was the first demonstration of interspecies HGT between these species (Colon et al., 1971). In the post-genomic era, we now know SDSE to be polylysogenic. Of the small number of genomes thus far sequenced the number of phage present range from one in SDSE RE378 to six in GGS_124 and AC-2713 (Watanabe et al., 2013) demonstrating their importance in promoting genetic diversity in the species. Molecular epidemiological studies have shown GAS-related superantigen genes to be absent or very rare in SDSE (Sachse et al., 2002; Kalia and Bessen, 2003; Davies et al., 2007; Tsai et al., 2013; Lo and Cheng, 2014) suggesting bacteriophage mediated HGT events between GAS and SDSE to be rare.

The first integrative conjugative element (ICE) in SDSE was described by Davies et al. (2009). ICE*Sde*3396 is a one of a group of related ICEs whose members also include ICE*Sa*2603 in GBS, ICE*Sde*32457 in *Streptococcus suis* (Palmieri et al., 2011, 2012) and a second ICE*Sde*3396-like element in SDSE 5580 (Palmieri et al., 2013). These ICEs have a common integration site in the *rplL* gene and share common genes involved in excision, transfer, and integration into recipient genome. Each also harbors a set of accessory genes that impart unique phenotypic features such as resistance to metals, or antibiotic resistance. These accessory regions are mosaic in nature and are likely to be the result of multiple recombination events. The closest homologs of these accessory genes can be found in different streptococcal species, and even non-streptococci. *In vitro* transfer of representative ICEs from this group to multiple recipient species, including GAS, *S. agalactiae*. SDSE and *S. pneumoniae* has been demonstrated by different groups (Davies et al., 2009; Palmieri et al., 2013). Based on this data it is likely that ICEs are the major source of novel genes in the SDSE population. Recently Palmieri et al. (2013) also demonstrated that the ICE*Sde*3396-element was able to mobilize and transfer a non-self-transmissible plasmid containing an erythromycin resistance gene from SDSE to other streptococcal species. This mechanism appears to be novel in the streptococci, and could explain how similar plasmids are transferred in other streptococci. An unrelated ICE, region of difference 2 (RD2), has also been identified in SDSE (Sitkiewicz et al., 2011). RD2 was first identified in GAS and is also similar to sequences in *S. agalactiae* (Green et al., 2005; Beres and Musser, 2007). RD2 carries several genes encoding putative surface proteins, including R28. R28 is involved in GBS binding to host vaginal cells. As RD2-positive GAS *emm*-types are also over-represented in puerperal infections the implication is that acquisition of RD2 has altered the pathogenesis of isolates within these *emm*-types.

## CONCLUSION

Our knowledge of the population structure, molecular epidemiology and pathogenic mechanisms of SDSE are lacking when compared to GAS and other pathogenic streptococci. However, it is apparent that HGT and recombination play an important role in generating genetic diversity within the species. New phenotypic repertoires associated with HGT may occasionally give rise to clones with increased fitness that could conceivably become dominant strains, or have increased virulence. The scale of HGT varies from replacement of individual domains within a gene to large MGE-associated transfers. While both bacteriophage and ICEs contribute to genetic diversity, current evidence indicates that it is ICEs that provide most novel genes to the population. In the future, comparative genomic sequencing of large numbers of SDSE isolates will provide a much greater depth to our understanding of these events, and how this bacterium has, and continues to evolve.

### Conflict of Interest Statement

The authors declare that the research was conducted in the absence of any commercial or financial relationships that could be construed as a potential conflict of interest.
